# Within-host Evolution of Segments Ratio for the Tripartite Genome of *Alfalfa Mosaic Virus*

**DOI:** 10.1038/s41598-017-05335-8

**Published:** 2017-07-10

**Authors:** Beilei Wu, Mark P. Zwart, Jesús A. Sánchez-Navarro, Santiago F. Elena

**Affiliations:** 1 Instituto de Biología Molecular y Celular de Plantas (IBMCP), Consejo Superior de Investigaciones Científicas-Universidad Politécnica de Valencia, Valencia, Spain; 2 Instituto de Biología Integrativa de Sistemas (I2SysBio), Consejo Superior de Investigaciones Científicas-Universitat de València, Valencia, Spain; 3 The Santa Fe Institute, New Mexico, USA; 4 Institute of Plant Protection, Chinese Academy of Agricultural Sciences, Beijing, China; 5 Institute of Theoretical Physics, University of Cologne, Cologne, Germany

## Abstract

The existence of multipartite viruses is an intriguing mystery in evolutionary virology. Several hypotheses suggest benefits that should outweigh the costs of a reduced transmission efficiency and of segregation of coadapted genes associated with encapsidating each segment into a different particle. Advantages range from increasing genome size despite high mutation rates, faster replication, more efficient selection resulting from reassortment during mixed infections, better regulation of gene expression, or enhanced virion stability and cell-to-cell movement. However, support for these hypotheses is scarce. Here we report experiments testing whether an evolutionary stable equilibrium exists for the three genomic RNAs of *Alfalfa mosaic virus* (AMV). Starting infections with different segment combinations, we found that the relative abundance of each segment evolves towards a constant ratio. Population genetic analyses show that the segment ratio at this equilibrium is determined by frequency-dependent selection. Replication of RNAs 1 and 2 was coupled and collaborative, whereas the replication of RNA 3 interfered with the replication of the other two. We found that the equilibrium solution is slightly different for the total amounts of RNA produced and encapsidated, suggesting that competition exists between all RNAs during encapsidation. Finally, we found that the observed equilibrium appears to be host-species dependent.

## Introduction

The highest level of physical organization of the genome is the division of the hereditary material into multiple segments. Genome segmentation is a ubiquitous feature of eukaryotes, with nuclear chromosome numbers covering an enormous range: from 2*n* to 630*n* ^1, 2^. In contrast, bacteria and archaea typically have a single chromosome^3^. Although many viruses also have a single genome segment, in some species the genome has been partitioned into multiple segments^4–6^. Whereas most viruses package multiple segments into a single virus particle (*e.g*., reovirus and orthomyxovirus), some plant and fungal viruses package each segment into a separate virus particle, a property known as multipartition. By contrast, the only multipartite animal viruses are the single-stranded DNA bidensoviruses infecting silkworms^7^ and the very recently discovered single-stranded positive-sense RNA virus in mosquitoes related to the *Flaviviridae* ^8^. In the extreme case, plant nanoviruses have up to eight DNA genome segments plus several satellite-like segments packaged up into different viral particles, although not all segments must enter a cell to cause infection^4^. For multipartite RNA viruses, the number of segments is typically lower, ranging from two to five. It is thought that all genome segments must enter the same cell to produce all the RNAs and proteins that are required to complete the process of infection and release the progeny that will infect new cells and transmit to new individual hosts^5, 6^.

The evolution of segmented genomes revolves around tradeoffs between potential costs and benefits inherent to different genome architectures. An obvious cost of multipartition is the necessity of coinfecting cells with at least one particle of each kind to ensure the presence of at least one copy of each segment, a cost that increases with the number of segments and particles^9^. All else being equal, an equimolecular composition of particles would maximize the probability of initiating the infection of a host cell successfully. Deviations from this situation would increase the cost of multipartition. Another potential cost of genome segmentation would be the breakage of co-adapted groups of genes during coinfection with several strains of the virus^10^. Several advantages have been proposed to compensate for these costs: (*i*) for the high mutation rates of most RNA viruses, smaller segments are more likely to be copied without errors than larger segments^11^, (*ii*) smaller genomic segments should be replicated faster^12^, (*iii*) segmentation favors genomic reassortment and thus increases genetic variability by rapidly bringing together beneficial mutations that have occurred in different lineages^13, 14^, minimizing the effect of clonal interference^15^ and speeding up the rate of adaptation, (*iv*) encapsidation of smaller genomes results in enhanced capsid stability^16^, (*v*) particularly in the case of plant viruses, smaller capsids would facilitate trafficking throughout the size-limiting plasmodesmata^17^, and (*vi*) segmentation represents an efficient yet simple way to control gene expression by regulating gene copy numbers^18^.

Starting on the mid-seventies, a number of publications have addressed different aspects of the replication and regulation of gene expression of plant multipartite viruses, especifically for members of the *Bromoviridae* family such as *Brome mosaic virus* (BMV) and *Cowpea chlorotic mottle virus* (CCMV). Many interesting conclusions were drawn in these studies, but particularly relevant for the problem of the evolution of multipartite virus are: (*i*) the ratio of RNA segments varies among closely related viral species (BMV and CCMV)^19^ and even among different BMV isolates^20^. (*ii*) The relative abundances of genomic segments for a particular bromovirus species varies among host species^21, 22^. (*iii*) Mutations in coding and noncoding sequences of different genomic segments have a profound impact on the accumulation of the other segments^23–27^.

Sicard *et al*. monitored the frequency of the eight single-gene-encoding segments, made of circular DNA, that constitute the genome of the nanovirus *Faba bean necrotic stunt virus* (FBNSV) during infection of single host plants^18^. They observed that regardless of the initial ratio of segments in the inocula, the ratio of segments always evolved towards a constant composition that the authors designated as the “setpoint genome formula” (hereafter referred as *SGF*), which did not represent an equimolecular mixture of genomic segments. They also found that the *SGF* corresponds to a state of maximal viral accumulation and of enhanced symptoms, thus suggesting that segmentation has evolved as a mechanism to regulate gene expression. Finally, in agreement with the bromoviruses results, they also found that the exact stoichiometry of the *SGF* depends on the host plant species. FBNSV, although being a well-suited model system for addressing questions related to genome segmentation and multipartition, is not very representative of most multipartite plant viruses: most are RNA viruses and have a lower number of genome segments.

As mentioned above, the molecular biology of multipartite RNA viruses has been extensively studied^19–27^, although it is not known for these viruses whether an *SGF* exists and whether it is evolutionarily stable. In other words: we still miss experimental evidences to support or reject some of the evolutionary genetic mechanism brought forward to explains the evolution of multipartition in RNA virus populations^9, 11–14^. If no stable *SGF* exists, or if multiple stable equilibria are possible, genome segment ratios could be in a state of perpetual flux, and some hypotheses for the advantages of segmentation would need to be discarded or revisited. Similarly, if different *SGF* exist in different host species, this would suggest that segmentation can facilitate adaptation by rapidly altering the proportion of RNAs –and possibly their expression– in a mutation-independent manner^18^.

The aim of our study is threefold. First, we sought to explore whether a *SGF* also exists for a prototypical multipartite RNA virus. Second, we also set out to determine the effect of host species on the stoichiometry of the *SGF*. Third, we also propose a novel analytical and computational framework, of universal applicability, to the evolutionary analysis of the abundance of any number of segments in segmented viral genomes. To tackle these questions, we have chosen *Alfalfa mosaic virus* (AMV; genus *Alfamovirus*, family *Bromoviridae*), whose genome is composed of three single-stranded positive-sense RNA molecules (RNA1, RNA2 and RNA3). Briefly, RNA1 and RNA2 encode proteins essential for replication (P1 and P2) while RNA3 encodes for the movement (MP) and coat (CP) proteins, the latter being translated from a subgenomic RNA4 (sgRNA4) produced by transcription of the negative-sense strand of RNA3^28^. Our results show that an evolutionary stable but host-species-dependent *SGF* occurs for this prototypical plant RNA virus.

## Results

### Determination of the *SGF* for AMV

**Figure 1:**
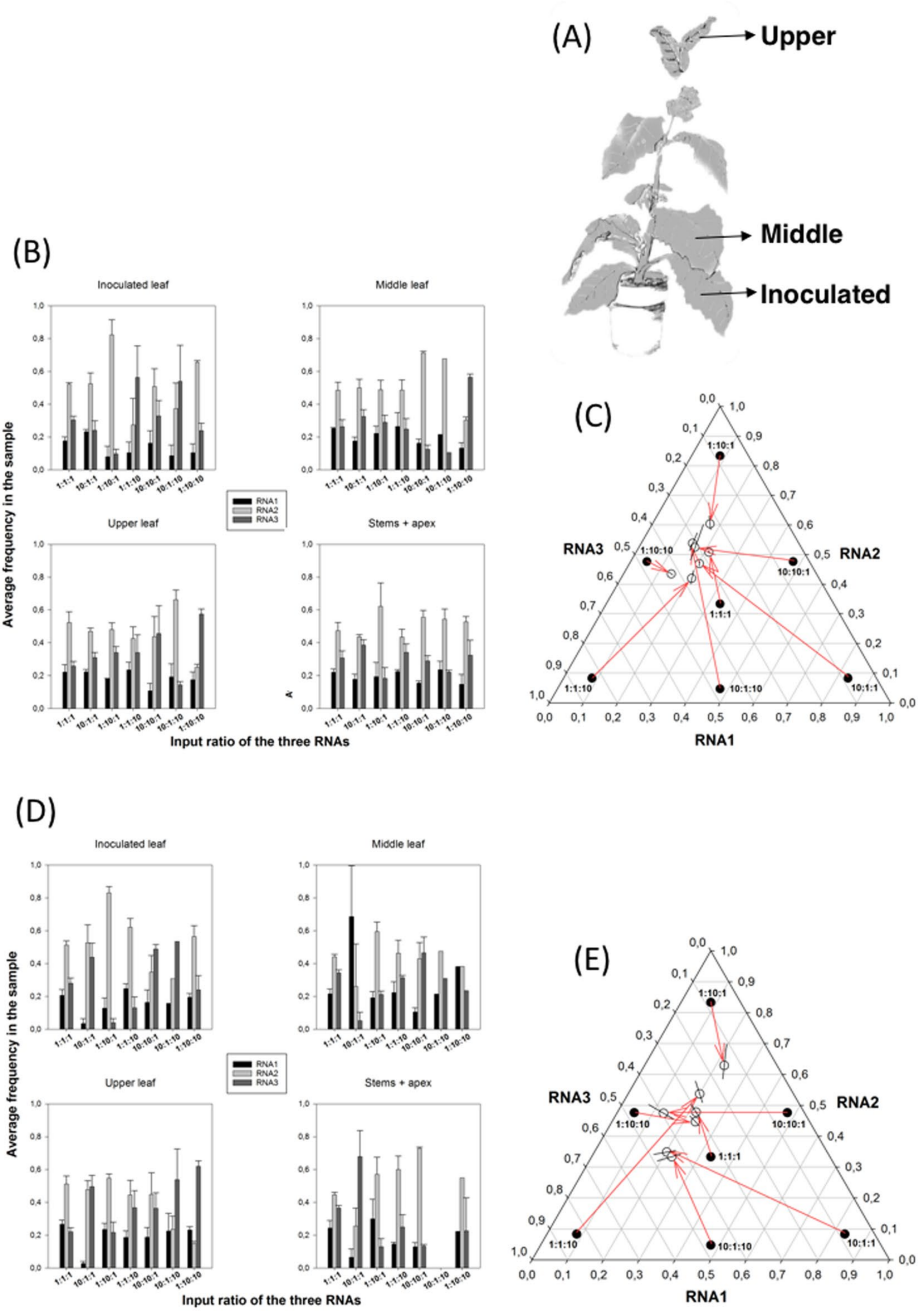
Effect of differences in the input ratio of RNA segments on the outcome of AMV infection. (**A**) Scheme of the sampling process, with indication of the three leafs sampled. (**B**) Experimental determinations (by RT-qPCR) of the frequency of each one of the three RNA segments in samples of total RNA for different input ratios. Bars represent the mean of *n* = 3 plants; error bars represent ±1 SEM. (**C**) Normalized frequency ternary plot showing the estimated *SGF*. Solid circles represent the indicated inoculation rates. Open circles show the marginal mean estimates of relative ratios at the end of the experiment corresponding to each input ratio. Lines crossing the open circles represent the 95% Cis. Red arrows connect initial and final ratios. (**D**) As in (**B**) but determined for encapsidated RNAs. (**E**) As in (**C**) but for encapsidated RNAs.

We first set out to determine whether an *SGF* exists for AMV, and if so to determine its characteristics. To this end we performed inoculation experiments with a range of RNA1, RNA2 and RNA3 ratios. *Nicotiana benthamiana* plants were mechanically inoculated with a constant amount of RNA (1 μg) but varying proportions of the three segments as detailed in the Methods section. The seven ratios employed were 1:1:1, 10:1:1, 1:10:1, 1:1:10, 10:10:1, 10:1:10, and 1:10:10. If a *SGF* exists, then we expect that these different combinations will all evolve towards it as infection progresses. We sampled different tissues (Fig. 1A) at different stages of infection and estimated the abundance of each RNA on the samples by RT-qPCR. Preliminary control experiments confirmed the specificity of each set of primers as well as their almost identical and high amplification efficiencies. Two types of RNA samples were prepared from each tissue: total RNA and encapsidated RNA from purified viral particles. The first sample represents the total amounts of RNA1, RNA2 and RNA3 synthetized during infection (the averages from *n* = 3 different plants are shown in Fig. 1B) whereas the second sample represents the amount of each RNA that has been encapsidated (the averages from *n* = 3 different plants are shown in Fig. 1D) and thus is expected to be the relevant figure in terms of horizontal virus transmission. Table 1A shows the results of the multivariate analysis of variance (MANOVA) analysis for the RNA frequencies from total RNA extractions fitted to equation (1). This equation, described in detail in the Methods section, relates all three experimental factors with the observed RNA segments frequencies: the RNA mixtures inoculated (*M*), the replicate plants inoculated with each RNA mixture (*P*) and the different tissues sampled (*S*). All three factors contribute in a highly significant manner to the observed variability in RNA segments frequency (in all cases *P* < 0.001). The resulting relative accumulations of RNA1, RNA2 and RNA3 differ among the seven input mixtures (factor *M*). Overall, plants inoculated with the same mixture also differ in the estimated output mixtures (factor *P* nested within factor *M, P*(*M*)). An overall difference also exist among tissue samples from plants inoculated with the same RNA mixture (factor *S* nested within factor *M*, *S*(*M*)). Finally, significant differences exist among the four sample types taken from each one of the three different plants inoculated with the same mixture of RNAs (factor (*P* × *S*)(*M*)).

**Table 1:** Results of the MANOVA analysis for the RNA frequencies estimated from total and from virion RNA extractions in experiments with variable input ratios done in *N. benthamiana*.

Effect	Wilk’s Λ	*F*	Hypothesis *df*	Error *df*	*P*	Power	${{\boldsymbol{\eta }}}_{{\boldsymbol{P}}}^{2}$
(A) Total RNA							
Intercept	0.002	16600.650	3	76	<0.001	1	0.999
*M*	0.026	31.742	18	215.446	<0.001	1	0.705
*P*(*M*)	0.015	16.813	42	226.218	<0.001	1	0.754
*S*(*M*)	0.003	21.563	63	227.701	<0.001	1	0.855
(*P* × *S*)(*M*)	0.008	8.224	111	229	<0.001	1	0.799
(B) Virion RNA							
Intercept	0.027	754.120	3	62	<0.001	1	0.973
*M*	0.325	4.760	18	175.848	<0.001	1	0.312
*P*(*M*)	0.499	1.162	42	184.687	0.248	0.958	0.207
*S*(*M*)	0.126	2.955	63	185.911	<0.001	1	0.499
(*P* × *S*)(*M*)	0.186	1.675	84	186.356	0.002	1	0.429

The model fitted is shown in equation (1). *M*: inoculated mixture of RNA segments; *P*(*M*): replicate plant inoculated with each mixture; *S*(*M*): type of sample from a plant inoculated with a given mixture; and (*P* × *S*)(*M*): interaction term between sample type and replicate plant inoculated with a given mixture *M*.

However, a significant *P*-value tells nothing about the *magnitude* of the effect that a factor has on the measured variables; a small effect may still be significant from an statistical point of view, while being too small to be biologically relevant. To assess the magnitude of effects we used the ${\eta }_{P}^{2}$ statistic. Table 1A shows that significant differences exist among plants inoculated with the same mixture (*P*(*M*) term in equation (1) in the Methods section) and among equivalent samples from different plants ((*P* × *S*)(*M*) term in equation (1)) and that the effect associated with these two factors is large (${\eta }_{P}^{2}$ = 0.754 and ${\eta }_{P}^{2}$ = 0.799, respectively). In statistics, ${\eta }_{P}^{2}\,$> 0.15 is usually considered as a large effect; all values reported in Table 1 are far larger than this threshold. These differences appear as an unavoidable consequence of the stochastic events that take place during inoculation of different plants as well as during the progression of infection (*e.g*., bottlenecks during cell-to-cell and systemic movement of viral particles^29^). Despite these differences, significant effects have been detected for the other factors.

The largest effect ${\eta }_{P}^{2}$ = 0.855 is associated to differences among samples (*i.e*., tissues; *S*(*M*) term in equation (1)). Differences among samples may result from a complex combination of factors. To mention a few, not necessarily being exhaustive, (*i*) different tissues being colonized by different subpopulations of RNAs simply by chance (*i.e*., bottlenecks and founder effects^29^), (*ii*) different tissues being at different developmental stages may impose differences in susceptibility to AMV infection, (*iii*) variability in the antiviral defense status of new tissues compared to old ones (*e.g*., antiviral RNAi-mediated response^30^), and even (*iv*) genetic differences in the composition of viral populations at different stages in their within-host evolution as a consequence of interplay between mutation and variable selection pressures on different tissues (*i.e*., the quasispecies nature of AMV)^31^. The weakest effect, ${\eta }_{P}^{2}$ = 0.705, corresponds to differences among inoculation ratios (*M* term in equation (1)), though it can still be considered as a very strong effect.

The grand mean estimate for the relative frequencies corresponds to a *SGF*_*total*_ of 1.00:2.88:1.87 (±1 SEM: 0.00:0.24:0.22). Figure 1C is a normalized ternary plot showing the marginal average of output ratios estimated for each input ratio. Regardless of the initial conditions (included in the plot to illustrate the coordinates of the different starting points), after infection all RNA populations tend to a rather limited region of the possible space of solutions, which contains the *SGF*_*total*_ ratio.

Table 1B shows the results from the MANOVA analysis run for the RNA frequencies estimated from encapsidated RNAs fitted to equation (1). The only difference with the results just reported for the total RNAs is the lack of differences among replica plants (term *P*(*M*) in equation (1); *P* = 0.248). The magnitude of significant effects is ${\eta }_{P}^{2}\ge$ 0.312, which is smaller than found for the case of total RNA but still considered as large (>0.15). In this case, the grand mean estimated for relative frequencies corresponds to a *SGF*_*encap*_ of 1.07:2.55:1.75 (±1 SEM: 0.07:0.39:0.26), a value that does not differ from the ~1:3:2 reported above for total RNAs, and in both cases shows that RNA2 is the most abundant one. As above, Fig. 1E summarizes the evolution of segments ratio from the input mixture to the average values obtained at the time of analyzing the encapsidated RNAs extracted: values converge to a particular region of the space that contains the *SGF*_*encap*_ value.

Overall, the results summarized in Fig. 1C and E suggest the existence of an attractor region in the frequencies phase diagram to which RNA populations converge after infection. In the following section, we will explore whether (*i*) this equilibrium is driven by frequency-dependent selection (FDS) and (*ii*) it is stable^32^.

### AMV *SGF* is driven by FDS to a stable equilibrium

**Figure 2:**
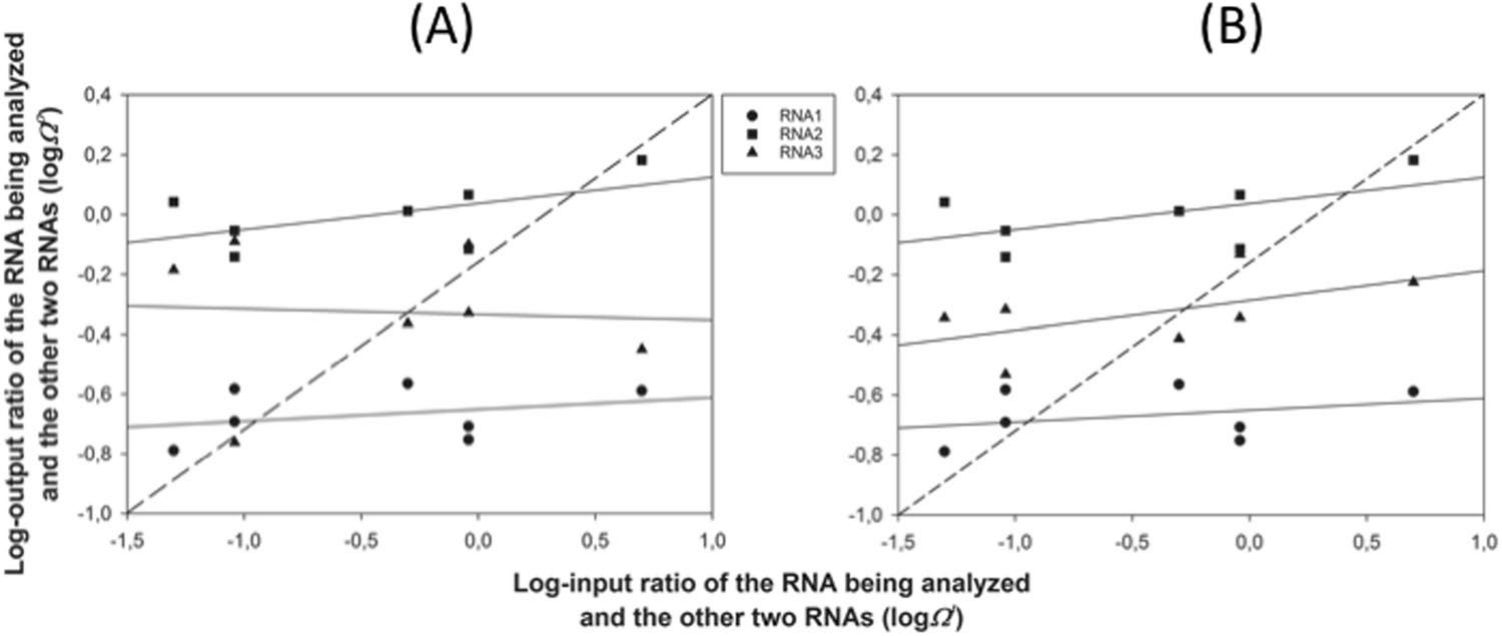
Graphical analysis of FDS as a mechanism to explain the relative abundance of the three RNA segments. (**A**) Abundances estimated from total RNA samples. (**B**) Abundances estimated from RNA extracted from viral particles. The dashed line corresponds to the null hypothesis of no-FDS. The continuous lines show the best fitting linear model to the relative abundances of each RNA.

Figure 2 shows the graphical analysis of FDS as a driving force of the observed *SGF*. Marginal mean frequency data shown in Fig. 1C and E for each RNA segment were transformed into relative abundances as described in the Methods section and then log-transformed. The plots show the output log-relative abundances as a function of the input log-relative abundances (the seven inoculation mixtures). Figure 2A shows the results for the RNA segments quantified in the total RNA samples (from Fig. 1C). The dashed line represents the expectation under the null hypothesis of no-FDS^32^. For each RNA, we computed the output and input log-relative abundance and plotted them (different symbols). First, the data were fitted to a set of models with increasing number of parameters, but in all cases the linear regression was the best-fitting model (shown as solid lines). In all three cases, statistical significance of the FDS is tested by the deviation of the slope of the linear regression from 1 (the dashed diagonal). All three regression lines in Fig. 2A have a slope significantly less than 1 (*t*_5_ ≥ 6.964, *P* ≤ 0.001). The analysis of Fig. 2A provides additional information of considerable biological interest, *i.e*., whether the FDS is linear or not, how strong it is, and if an equilibrium point exists. A point of equilibrium occurs if and where the regression line crosses the diagonal^32^. At such point the focal RNA segment frequency is at the same frequency as expected in the absence of FDS. In all three cases, the slope is less than one, meaning that the equilibrium is evolutionarily stable; the system evolves towards an equilibrium *SGF*_*total*_ that is stable against random perturbations of any of the three RNA components. For instance, perturbations may be associated with the inoculation process or by bottlenecks inherent to systemic movement and colonization of new growing tissues in the apical meristem^29^.

Figure 2B presents the graphical analyses of FDS for the encapsidated RNA ratios shown in Fig. 1E. The conclusions are qualitatively the same as those described in the previous paragraph for the total RNA samples: all three relationships are linear, with slopes significantly less than 1 (*t*_5_ ≥ 13.610, *P* ≤ 0.001) and thus the *SGF*_*encap*_ also corresponds to an evolutionarily stable equilibrium. Notice that in the case of encapsidated RNAs, the three curves show similar positive slopes while in the case of the total RNAs the curves for RNA1 and RNA2 are similar in slope and both positive wherease the curve for RNA3 is negative.

To further characterize the nature of this FDS, we have analyzed the particular relationship between the marginal mean abundances of RNAs in both types of samples, from total and encapsidated RNAs. To do so, we have computed partial correlation coefficients among RNA abundances using as control variables the input ratios (*M* in equation (1)), plant replicate (*P* in equation (1)) and tissue sampled (*S* in equation (1)). Table 2 shows the hemi-matrix of correlations (notice that the matrix is symmetrical and thus the upper half has been removed). Focusing first in the quantifications from the total RNA extractions, we found that the synthesis of RNA3 negatively correlates with the production of both RNA1 and RNA2, while the levels of RNA1 and RNA2 production do not affect each other (lack of significant correlations). Looking now at the correlations between encapsidated RNAs, we found that all are negatively correlated with each other, thus suggesting that they compete for available capsids, which should then become a limiting factor. Finally, looking at correlations between total and encapsidated RNAs (non-gray cells in the hemi-matrix), with exception of RNA1, positive correlations exist between the amount of total and encapsidated RNAs (although the correlation for RNA3 becomes non-significant after accounting for multiple tests of the same hypothesis). RNA2 and RNA3 seem to strongly compete for encapsidation: the more RNA2 produced, the less RNA3 encapsidated and *vice versa*. However, RNA1 does not seem to be involved in this competition.

**Table 2:** Results from the partial correlation analyses among abundances of different RNA segments.

		Total RNA extraction			Virion RNA extraction	
		RNA1	RNA2	RNA3	RNA1	RNA2
Total	RNA2	*r* = 0.024, *P* = 0.835				
	RNA3	*r* = −0.445, *P* < 0.001^*^	*r* = −0.895, *P* < 0.001^*^			
Virion	RNA1	*r* = −0.001, *P* = 0.991	*r* = −0.049, *P* = 0.661	*r* = 0.052, *P* = 0.645		
	RNA2	*r* = −0.021, *P* = 0.850	*r* = 0.319, *P* = 0.004^*^	*r* = −0.282, *P* = 0.011^*^	*r* = −0.425, *P* < 0.001^*^	
	RNA3	*r* = −0.020, *P* = 0.863	*r* = −0.278, *P* = 0.012^*^	*r* = −0.238, *P* = 0.032	*r* = −0.418, *P* < 0.001^*^	*r* = −0.644, *P* < 0.001^*^

All tests have 79 *df*. Asterisks indicate cases significant after the Holm-Bonferroni correction of multiple tests of the same null hypothesis.

### AMV *SGF* varies among host species

**Figure 3:**
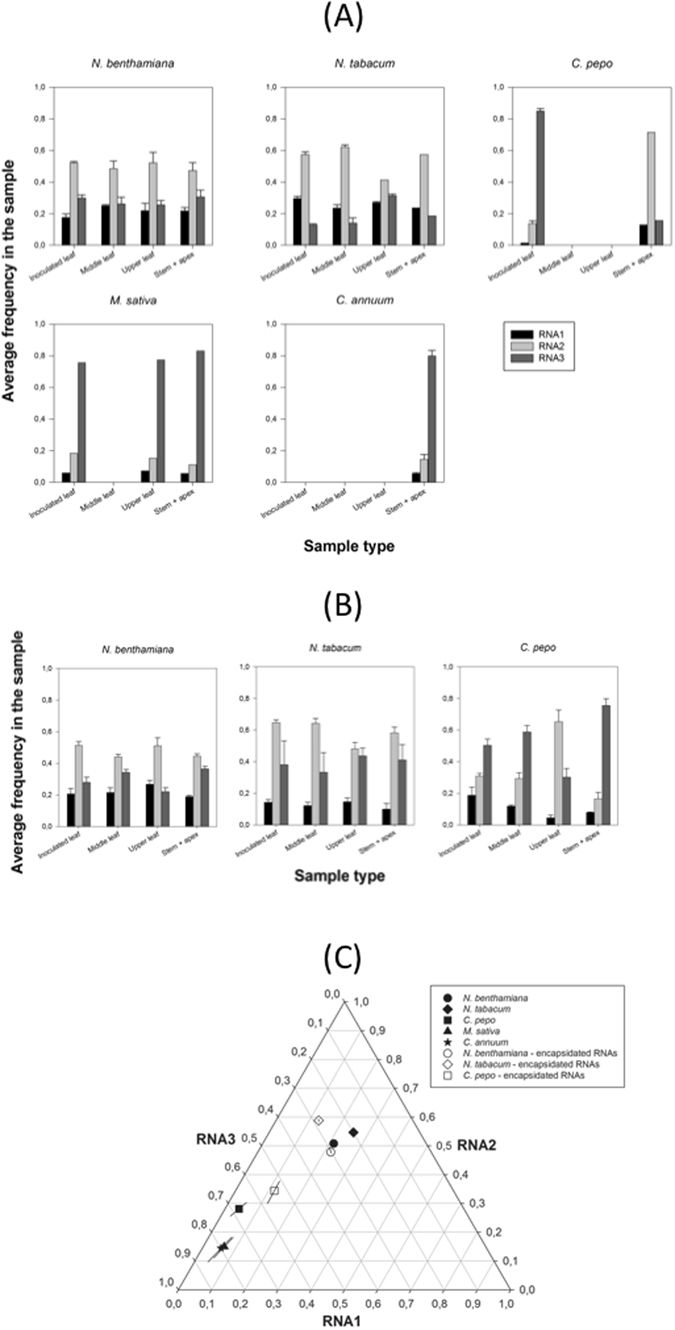
Effect of host species in the accumulation of each genomic segment of AMV. (**A**) Experimental determinations (by RT-qPCR) of the frequency of each AMV genomic in total RNA extractions from five different host species. Bars represent the mean of *n* = 3 plants per hsot species; error bars represent ±1 SEM. (**B**) As in (**A**) but determined for encapsidated RNAs. No data are available for *C. annuum* and *M. sativa*. (**C**) Normalized frequency ternary plot showing the effect of host species in the estimated *SGF*. Solid symbols represent the marginal mean frequencies estimated from total RNA samples. Open symbols represent the marginal mean frequencies estimated from virion RNA samples. Lines crossing symbols represent 95% CIs.

Next, we explored to which extent the host species determines the value of *SGF*. To do so, we inoculated five different susceptible hosts (*N. benthamiana*, *Nicotiana tabacum*, *Cucurbita pepo*, *Medicago sativa*, and *Capsicum annuum*) with a 1:1:1 mixture of the genomic RNA segments and evaluated the output frequency of each segment 7 days post inoculation days (dpi) for *N. benthamiana* and 12 dpi for the rest of species, following the same sampling scheme than in the experiments previously described. Frequency data are shown in Fig. 3A (averages from *n* = 3 different plants). These data were fitted to equation (2) in Methods using MANOVA and the results from this analysis are shown in Table 3. In case of segment frequencies in the total RNA extraction, all factors had a highly significant effect, with magnitudes being in all cases ${\eta }_{P}^{2}\ge$ 0.483 (Table 3A). There is great variation in the segments ratio among host species (solid symbols in Fig. 3C), although the estimates for both *Nicotiana* spp. remain closer among them than they are relative to the other species analyzed. The grand mean value *SGF*_*total*_ across hosts is 1.05:3.36:9.04 (±1 SEM: 0.05:0.84:3.27), a value that sharply contrasts to the above stable equilibrium value found for *N. benthamiana* (~1:3:2) due to the larger bias in the synthesis of RNA3 that characterizes the infection of *C. pepo* (1.00:6.70:16.11 (±1 SEM: 0.39:1.86:3.42)), *M. sativa* (1.00:2.37:12.45 (±1 SEM: 0.27:0.70:1.87)) and *C. annuum* (1.00:2.61:14.35 (±1 SEM: 0.35:0.96:2.78)).

**Table 3:** Results of the MANOVA analysis for the RNA frequencies estimated from total and from virion RNA extractions in experiments with 1:1:1 input ratio done in five different host species.

Effect	Wilk’s Λ	*F*	Hypothesis *df*	Error *df*	*P*	Power	${{\boldsymbol{\eta }}}_{{\boldsymbol{P}}}^{2}$
(A) Total RNA							
Intercept	0.002	5932.782	3	39	<0.001	1	0.998
*E*	0.004	61.443	12	103.456	<0.001	1	0.842
*P*(*E*)	0.139	2.828	39	116.235	<0.001	1	0.483
*S*(*E*)	0.035	11.953	21	112.537	<0.001	1	0.675
(*P* × *S*)(*E*)	0.017	6.014	57	117.111	<0.001	1	0.743
(B) Virion RNA							
Intercept	0.007	1631.200	3	32	<0.001	1	0.994
*E*	0.038	44.242	6	64	<0.001	1	0.806
*P*(*E*)	0.373	2.108	18	90.995	0.011	0.957	0.280
*S*(*E*)	0.059	5.714	27	94.099	<0.001	1	0.611
(*P* × *S*)(*E*)	0.215	1.352	48	95.970	0.106	0.976	0.401

The model fitted is shown in equation (2). *E*: plant species; *P*(*E*): replicate plant from species *E*; *S*(*E*): type of sample from a plant of a given species; and (*P* × *S*)(*E*): interaction term between sample type and replicate plant from species *E*.

Frequency data obtained from encapsidated RNAs are shown in Fig. 3B. Quantifications obtained for encapsidated RNAs from *M. sativa* and *C. annuum* were not different from negative controls and thus were not considered for the following analyses. In case of segment frequencies in the encapsidated RNAs, the only not significant factor was the interaction between plant replicate and type of sample ($(P\times S)(E)$ term in equation (2)) (Table 3B), although highly significant differences exist among host species. In this case, the grand mean value *SGF*_*encap*_ across hosts is 1.00:3.24:2.74 (±1 SEM: 0.00:0.71:0.97), which also differs from the stable equilibrium value found for *N. benthamiana* but to a lesser extent; *e.g*., for *C. pepo* 1.00:2.95:4.62 (±1 SEM: 0.12:0.54:0.44). Figure 3C (open symbols) also shows that estimates obtained from total and encapsidated RNA extractions render values that are close in the normalized ternary plot, thus showing a good correlation among them. Therefore, we can conclude that the segments ratio at 12 dpi strongly depends on the host species which is being infected, which suggests the *SGF* is host-species dependent.

### Total RNA production is maximized at the *SGF*_*total*_

We have observed that *SGF*_*total*_ is maintained by a FDS mechanism, and that the actual value taken by the *SGF*_*total*_ depends on the host wherein AMV replicates. Has *SGF*_*total*_ been optimized in each host by natural selection to maximize the total accumulation of the three genomic RNAs? To tackle this question, we have computed a partial correlation coefficient between the total RNA accumulation (summing up the accumulations of the three RNA segments) and the Euclidean distance (other multivariate distances have been tested, with identical results) from the *SGF* values obtained for each individual sample (*i.e*., a particular tissue from a given plant from each host species) and their corresponding evolutionarily stable *SGF*_*total*_ estimates using as control variables the host species (*E* in equation (2)), input ratios (*M* in equation (1)), plant replicate (*P* in equation (2)) and tissue sampled (*S* in equation (2)). The rationale behind this test is as follows: if *SGF*_*total*_ has been optimized to maximize the production of AMV genomic RNAs, then the farther from the evolutionarily stable *SGF*_*total*_ equilibrium the replicating AMV population, the lower the accumulation of genomic RNAs. Conversely, the closer to the equilibrium *SGF*_*total*_ a replicating viral population would be, the higher the accumulation of AMV RNAs. A low yet highly significant negative correlation exists between distance to the optimal *SGF*_*total*_ and total RNA accumulation (*r* = −0.249, 120 d.f., 1-tailed *P* = 0.003), thus backing up the hypothesis that RNA accumulation is maximal at the equilibrium *SGF*_*total*_. Notice that this test implicitly assumes that no defective genomes are being produced and hence every RNA molecule that has been counted would eventually contribute to successfully complete the infection process. If a fraction of the accounted molecules was defective, then the test would still be valid as far as the number of defective molecules for each of the three RNAs would distribute proportionally to their rate of production. If an RNA segment produces more defective copies than others, the validity of the test would be jeopardized.

By contrast, despite being negative as expected under the above hypothesis, the correlation observed between the distance to the evolutionary stable value of *SGF*_*encap*_ and the total amount of the three RNAs encapsidated was not significant (*r* = −0.133, 89 d.f., 1-tailed *P* = 0.105), suggesting that the strength of selection for encapsidation has been weaker than for replication or, alternatively, that selective constraints for encapsidation may differ among tissues and that by pooling together data from different tissues we have lost the statistical power to detect a significant correlation.

### Transgenic expression of P1 and/or P2 proteins disrupts *SGF*

**Figure 4:**
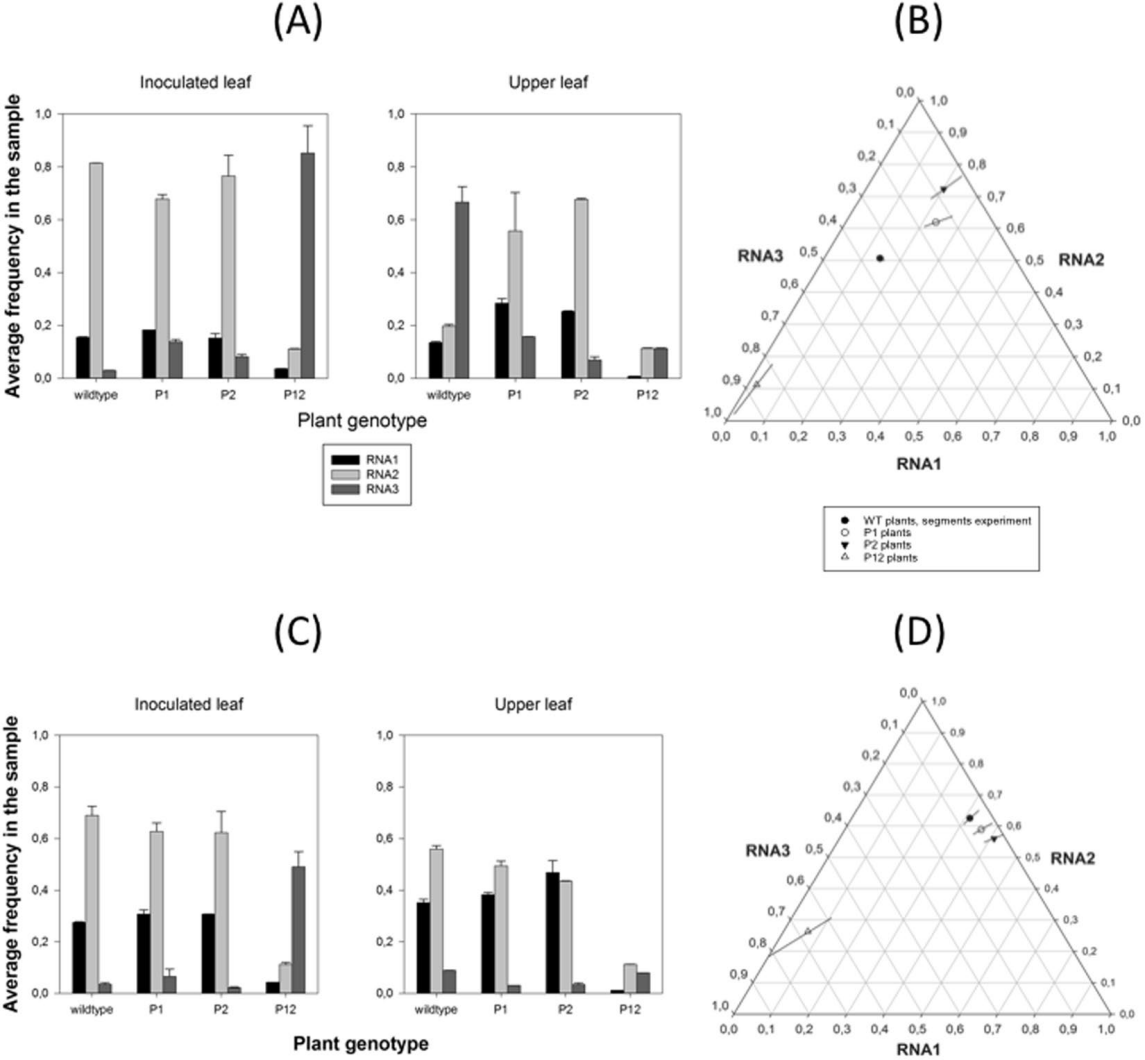
Accumulation of the three genomic segments of AMV on transgenic *N. benthamiana* plants that express viral proteins P1, P2 or both. (**A**) Experimental determinations (by RT-qPCR) of the frequency of each AMV genomic in total RNA extractions from five different host species. Bars represent the mean of *n* = 3 plants; error bars represent ±1 SEM. (**B**) Normalized frequency ternary plot showing the effect of transgenic expression of viral RNAs in the estimated *SGF*. (**C**) As in (**A**) but determined for encapsidated RNAs. (**D**) As in (**B**) but for encapsidated RNAs.

In the next set of experiments, we explored the effect on the segments ratio of constitutive overexpression of RNA1 and RNA2 encoding for proteins P1, P2 or both proteins by transgenes inserted in the plant. Our hypothesis is that by providing these proteins transgenically in a large excess, the *SGF* will be largely perturbed in a non-random manner. This last qualification is important, since we found that *SGF* represents an evolutionary stable equilibrium and thus small random perturbations will result in the system returning to this equilibrium. To test this hypothesis, we inoculated transgenic *N. tabacum* plants expressing P1, P2 or P1 and P2 (P12 plants)^33^ with a 1:1:1 input ratio of the three segments. As in all experiments described so far, we quantified the output segments frequencies in both total RNA extracts (data shown in Fig. 4A) and in RNA preparations from previously purified viral particles (data shown in Fig. 4C). These frequency data are then fitted to the MANOVA model shown in equation (3) in the Methods section. Table 4 shows the results of the analysis. Regardless the type of RNA sample analyzed, significant differences exist in the output frequencies among genotypes (term *E* in equation (3)). The magnitude of the effects associated with all factors is large (in all cases ${\eta }_{P}^{2}\ge$ 0.821 for estimates from total RNAs extraction and ${\eta }_{P}^{2}\ge$ 0.715 for estimates from encapsidated RNAs). The grand mean value estimated *SGF*_*total*_ is 1.55:5.58:10.94 (±1 SEM: 0.40:1.36:9.48), which departs from the stable equilibrium described above, yet with a large excess of RNA3. Paying attention to the situation in different transgenic plants, the marginal means are 1.58:4.19:1.00 (±1 SEM: 0.01:0.04:0.03) for P1, 2.68:9.53:1.00 (±1 SEM: 0.01:0.04:0.03) for P2 and 1.00:5.10:39.36 (±1 SEM: 0.01:0.04:0.03) for P12, suggesting large departures from the equilibrium *SGF*_*total*_ (Fig. 4B). Regarding encapsidated RNAs, the marginal *SGF*_*encap*_ means vary even widely: 7.20:11.71:1.00 (±1 SEM: 0.01:0.03:0.02) for P1, 13.60:18.58:1.00 (±1 SEM: 0.01:0.04:0.03) for P2 and 1.00:4.08:10.40 (±1 SEM: 0.01:0.04:0.03) for P12 (Fig. 4D). The grand mean value in this case is 6.7:11.11:3.35 (±1 SEM: 2.63:2.98:2.35).

**Table 4:** Results of the MANOVA analysis for the RNA frequencies estimated from total and from virion RNA extractions in experiments with 1:1:1 input rations done in wildtype and P1, P2 and P12 transgenic *N. benthamiana*.

Effect	Wilk’s Λ	*F*	Hypothesis *df*	Error *df*	*P*	Power	${{\boldsymbol{\eta }}}_{{\boldsymbol{P}}}^{2}$
(A) Total RNA							
Intercept	0.002	1114.421	3	6	<0.001	1	0.998
*E*	0.002	20.386	9	14.753	<0.001	1	0.879
*S*	0.095	19.062	3	6	0.002	0.996	0.905
*E* × *S*	0.006	12.063	9	14.753	<0.001	0.998	0.821
(B) Virion RNA							
Intercept	0.002	924.673	3	6	<0.001	1	0.998
*E*	0.003	16.705	9	14.753	<0.001	1	0.859
*S*	0.081	22.714	3	6	0.001	1	0.919
*E* × *S*	0.023	6.064	9	14.753	0.001	0.933	0.715

The model fitted is shown in equation (3). *E*: *N. benthamiana* genotype; *S*: sample type; *E* × *S*: plant genotype by sample type interaction term.

We have checked whether transgenic expression of RNA1 and/or RNA2 may trigger antiviral RNAi. To do so, we have analyzed the total accumulation of RNA1 and RNA2 in infected P1, P2 and P12 plants relative to the accumulation of these two viral RNAs in wildtype plants. If RNAi is at play, then RNA accumulation in the transgenic plants would be reduced relative to the accumulation in wildtype plants. These analyses show that RNA1 accumulates, on average, 54.14% less in P1 plants, 25.34% less in P2 plants and up to 99.66% less in P12 plants than in wildtype plants. Likewise, RNA2 accumulates, on average, 76.89% in P1 plants, 50.75% less in P2 plants and 99.66% less in P12 plants than in WT plants. Notice that the accumulation of RNA2 in P2 plants is less affected than accumulation in P1 plants, which is not fully explained by the operation of RNAi itself, and other mechanisms may at work as well. Therefore, antiviral RNAi is probably at play in the transgenic plants; alas it would be hard to fully separate its effect from the effect of a non-optimal segments ratio. In any case, whether or not antiviral RNAi may be turned on in these transgenic plants prior to virus inoculation, is not invalidating our conclusion that a strong non-random perturbation of the *SGF* does not allow the virus to return to the equilibrium condition. At best, these results illustrate that the magnitude of the perturbation results from the contribution of two opposite forces: mRNA transcription from the transgene and RNAi-mediated degradation of these mRNAs and the viral RNAs.

In conclusion, constitutive and high expression of viral RNA components from a transgene strongly perturb the system and take it far away from its stable equilibrium, which cannot be reached again because the constant input of RNA1 and/or RNA2. It is striking that in most cases, the *SGF*_*total*_ shows greater propensity for change than *SGF*_*encap*_. There are many mechanisms that could underlie this observation, although it suggests that there might be a tradeoff between within-host spread and virus particle yield per cell.

## Discussion

Here, we have explored the within-host evolution of the ratio of the three genomic segments of the multipartite plant virus AMV. We found that regardless the ratio used at inoculation, an evolutionary stable equilibrium is reached in which the three RNA segments are represented in a ~1:3:2 stoichiometric ratio, in *N. benthamiana*. The occurrence of a stable genome-segment formula has been dubbed as the setpoint genome formula, or *SGF*, by Sicard *et al*., who described within-host evolution towards a stable composition for a multipartite DNA virus, FBNSV^18^. Here, we have extended this observation to a second multipartite virus, which is more representative of the vast majority of multipartite viruses by virtue of being an RNA virus. We have observed that the ratio of encapsidated segments also represents an evolutionary stable equilibrium with a very similar composition: ~1:3:2. Related to these observations, we found that the production and encapsidation of the different segments are linked in a non-trivial manner, at least in *N. benthamiana* plants. We speculate that RNA1 and RNA2 cooperated during replication whilst competing with RNA3 (supported by the significant negative correlations between total amounts of RNA1 and RNA2 and of RNA3), and that all three RNAs competed with each other for the CP for encapsidation (supported by the significant negative correlations between the amount of all three encapsidated RNAs). With the only exception of RNA2, no positive correlation exists between the accumulation of total and encapsidated RNAs, suggesting that the process of encapsidation is not only determined by the accumulation of the corresponding RNAs. Interestingly, negative correlations exist between the total accumulation of RNA2 and encapsidated RNA3 and *vice versa* but not with total and encapsidated forms of RNA1. Although these inferences on the interactions between the genome segments are based on a correlation analyses, they do reflect a biologically relevant association between viral traits. However, we have not tested the underlying mechanisms, and do not know whether there is a direct causal link between both traits or the correlation is mediated by a third yet unknown factor. Future work will explore the mechanisms of these correlations.

We found that the *SGF* appears to be dependent on the host species, suggesting the involvement of host factors that differ among host species play a role in its regulation. In agreement with our findings, Ni *et al*. also found that the relative abundances of encapsidated and total RNA segments of BMV were also dependent on the host species^34^. Indeed, when the ratios of the three segments were followed during the progression of infection in two monocot hosts, they converge into stable *SGF*_*total*_ (1:2:3 for barley and 1:2:2 for wheat). However, at odds with our findings, these authors concluded that no relationship existed between the total RNAs produced and their relative encapsidation. Nonetheless, a significant correlation exists between the relative frequencies of total and encapsidated RNAs in wheat (Spearman’s *ρ* = 1, 2 d.f., *P* < 0.001) but not in barley (*ρ* = 0.400, 2 d.f., *P* = 0.600).

Multiple theories have been proposed to explain the existence of multipartite viruses, most commonly found in plants. The most recent and tantalizing proposal is that genome segmentation represents an efficient and rapidly adaptable way of regulating gene expression throughout manipulation of gene copy numbers^18^. As the changes in *SGF* observed here in alternative host species occurred within a narrow time window, multipartition might be advantageous for rapid adaptation to new hosts in a manner that is largely nucleotide-sequence independent, and therefore also mutation independent. Such an approach to adaptation could be especially advantageous in alternative hosts, where founder numbers may be small due to low infection probabilities and effective population sizes might also not be large initially due to poor replication. The hypothesis of segmentation as a mechanism to regulate gene expression has been proposed for segmented DNA viruses^6, 18^. Unfortunately, in the case of RNA viruses, it is not trivial to distinguish between RNA molecules that are serving as templates for replication from those that are serving as mRNAs at different stages during infection, therefore, we cannot directly test this hypothesis with our data. Additional experiments quantifying the number of RNA segments that either serve as mRNA, are encapsidated, or simply not used in any way, would be necessary for directly testing this possibility for a segmented RNA virus.

On the other hand, according to this hypothesis, a tight link must exist between the necessity of producing a given protein and the abundance of the RNA segment that encodes for it. At first glance, this hypothesis does not apply to AMV for two reasons. Firstly, as one may imagine that CP, necessary for producing infectious virions and encoded by the RNA3, would be required in larger numbers than the replicase complex, encoded by RNA1 and RNA2. However, it is important to recall at this point that CP is translated from a sgRNA4. We have not quantified the abundance of this subgenomic RNA. Interestingly, however, we observed that the ratio of RNA1 and RNA2 remains more or less constant in all experimental conditions tested (see below). Secondly, our observation of RNA2 accumulating more than RNA1 may suggest that P2 should also accumulate more than P1. Unfortunately, no quantitative data are available on the accumulation of AMV P1 and P2 in virus infected tissues. Comparing with other members of the *Bromoviridae* family, it has been shown that BMV and *Cucumber mosaic virus* (CMV) 1a protein accumulates to larger amounts than the corresponding 2a protein in purified replication complexes^35–37^, in spite that the *SGF*_*total*_ for BMV was 1:2:3 for barley and 1:2:2 for wheat, suggesting that either *trans* elements are controlling the translation of viral RNAs or that *cis*-acting elements may affect the efficiency of each RNA in recruiting ribosomes. In this sense, it has been observed that AMV CP enhances the translational efficiently of viral RNAs *in vivo* ^38^ via the interaction with the 3′ termini, which adopts two alternative structures for translation (a linear array of hairpins with high affinity for CP) and replication (a pseudo-knotted structure)^39^. A similar mechanism has been reported as regulator for translation of the replication complex proteins of BMV^40^. The assumption that the amount of protein expressed is always proportional to the amount of messenger RNA, although appealing, has been proven wrong. For example, during mixed phage infections of bacterial cells, increasing the number of genomic copies results in switches between lytic and lysogenic states and the concomitant production of viral proteins^41^. Indeed, in such instances the regulation of is an emerging property of the structure of regulatory networks rather than directly resulting from gene copy number^41^. Translation efficiency and RNA stability are inexorably linked^42^, further challenging simple interpretations of the effects of observed RNA levels on actual protein expression levels. Indeed, it would not be surprising that RNA stability and translational productivity of the three RNA segments of AMV may be affected by the actual host species.

We found significant differences between the *SGF* estimated from total RNA production and from encapsidated RNA. These results may stem in part from different interactions between the different 3′ untranslated regions of the RNA segments and the CP, and their effects on encapsidation^43^. Furthermore, selection may operate in distinct ways here, eventually resulting in an evolutionary tradeoff. On the one hand, within-cell selection on replication will result in a *SGF*_*total*_ that maximizes replication of the three RNA segments, likely by producing an optimal combination of RNAs and proteins, thus a segments ratio that would necessarily depart from the 1:1:1 as more proteins encoded by one segment are needed than proteins from other segments. This possibility is clearly supported by the negative correlation that we have observed between the total amount of genomic RNA produced and the distance to the *SGF*_*total*_. On the other hand, selection operating at the systemic movement and at the between-host levels will result in a *SGF*_*encap*_ that maximizes the probability of successful transmission. The *SGF*_*encap*_ values that maximize within-cell replication and transmission might be different, resulting in a tradeoff. However, our observations do not back up this possibility, as we have not observed the predicted negative correlation between total encapsidated RNAs and the distance to the *SGF*_*encap*_.

The mechanisms that may determine AMV’s *SGF* remain elusive. Probably the evolutionarily stable *SGF* results from complex molecular interactions between viral and host components, inextricably intertwined with viral population dynamics. Some evidences available in the literature may help to bring light into this complex question. For instance, results from transient expression experiments of proteins P1 and/or P2 revealed that replication of RNA1 and RNA2 depends on the presence of these proteins in *cis* and that, within infected cells, the replication of RNA1 and RNA2 is strictly coordinated through the encoded proteins rather than by RNA-RNA interactions^44^. This coordination may ensure the expression of proteins P1 and P2 in the correct ratio to form the replication complex. However, the replication of RNA3 is not linked to the replication of RNA1 and RNA2^45, 46^. In this sense, these interactions explain the results reported in Table 2, namely, the negative correlation observed between replication of RNA3 and production of RNA1 and RNA2: replication of both RNA1 and RNA2 is coupled and not interfering each other, while the replication of RNA3 must use the full replicase complex in *trans* and, thus, competes with the replication of RNA1 and RNA2. These observations lead to the prediction that the ratio between RNA1 and RNA2 could be constant (see below) but also suggest that the different RNAs of segmented viruses could be considered as independent molecules unless they replicate coordinately, with *cis* elements that constraint the accumulation of the corresponding viral RNAs. Independent of whether different viral RNAs are coupled or not, we observed that providing a specific viral protein in *trans* (P1, P2 or P12 transgenic plants) alters the AMV *SGF*, and thereby probably the relationship between accumulation, virulence and transmission. It is tempting to speculate that virus resistance mediated by the expression of viral proteins –normally the CP in transgenic plants– could be at least partially due to a drastic alteration of the genome segment ratio and the negative effects thereof on viral replication.

Regarding the results obtained from hosts other than *N. benthamiana*, first we must acknowledge a limitation of our experimental design: as we did not consider different starting ratios or multiple time points post inoculation, one could question whether virus populations have reached a stable *SGF*. Conservatively speaking, we can only conclude that at advanced stages of infections in all hosts (*i.e*., 12 dpi), the ratio of segments significantly differs among hosts and significantly departs from the one value estimated for *N. benthamiana*. This being said, we observed that the ratio between RNA1 and RNA2 remains constant (ca. 1 RNA1 molecule per 3 RNA2 molecules) probably due to the coordinated replications between both RNAs, as mentioned in the previous paragraph. The coordinated replication of both RNA1 and RNA2 may determine the ratio of both viral RNAs independently of the host species. In this sense, we observed that the ratio of both viral RNAs oscillated between 1:2–1:3. Even in the highly artificial situation in which one of the replicase components was constitutively provided in *trans* by the plant but the other necessarily was translated from viral RNAs (P1 or P2 plants), the ratio between both RNA1 and RNA2 was maintained between 1:2 (2:4:1 for P1) and 1:3 (3:9:1 for P2). This suggests that the RNA1, that encodes for the methyltransferase-helicase P1 protein, is actually the most limiting factor in the system. Protein P1 is required to form a complex with the P2 RNA-dependent RNA polymerase protein (encoded by RNA2), acting *in trans* for replication and transcription of RNA 2, RNA3 and sgRNA4. However, efficient replication of RNA1 may also require translation of RNA1 into P1 protein in *cis*, indicating a linkage between translation and replication of this segment, as shown for BMV^47, 48^. Only in the case of P12 plants, the ratio was completely altered between the three RNAs, probably due to the transgenic presence of the full replicase complex in *trans* (P12 plants) and the incapacity of the virus to modulate the transgenes or its expression. It is interesting to note the low accumulation of RNA3 in P1 and P2 plants but not in P12 plants, which is consistent with the observation that coordinated expression of BMV proteins 1a and 2a from RNA1 and RNA2, respectively, is also required to complete the synthesis of RNA3 in transgenic yeast cells^49^. Apparently, the system dynamically evolves to maintain the correct ratio between RNA1 and RNA2, to the detriment of the accumulation of the RNA3. Furthermore, the accumulation of RNA3 was significantly altered depending of the host species, indicating that the virus may use this RNA to accommodate its life cycle to the presence/absence of different host factors, for instance, the transcription factor promoting salicylic-dependent defense signaling response recently reported to interact with the AMV CP^50^.

All three AMV RNAs contain binding sites for the CP at the 3′UTR and bind it with an equal distribution between all viral RNAs^43, 51^. In solution, AMV CP occurs as dimers and these dimers are the building blocks of viral capsids^51^. N-terminal peptides of CP bind to the 39 nucleotides of the 3′UTR RNAs in a 2:1 stoichiometric ratio^52^. Binding of the CP to the 3′UTR also enhances translation of viral RNAs by mimicking the function of the host poly(A)-binding protein^53, 54^. Altogether, these evidences point to the idea of a CP with multiple functions that are critical at different steps of the virus infectious cycle. The results obtained in the present work support the idea that the RNAs are competing for the CP, and it is therefore a limiting factor that could be used for interventions aimed at controlling virus infection. In agreement with this result, it has been recently observed that AMV CP accumulated at the nucleus and nucleolus, an observation interpreted as a mechanism to control virus expression by the cytoplasmic/nuclear balance of CP accumulation^55^. The sequestering of the CP at the nucleus may represent a defensive strategy to control the virus cycle, but also the accumulation of the viral RNAs containing this open reading frame.

## Methods

### Host species and virus inoculation

Plants from the experimental hosts *C. annuum* L., *C. pepo* L., *N. benthamiana* Domin, *N. tabacum* L. cv. Samsun, and *M. sativa* L. were all mechanically inoculated with 5 μL of a mixture of 5′-capped transcripts corresponding to AMV strain 425 RNA1, RNA2 and RNA3 plus a few μg of purified AMV CP as described previously^56^. In addition, transgenic *N. tabacum* plants that express AMV polymerase proteins P1 (P1 plants) and P2 (P2 plants) or both (P12 plants)^33^, were also assayed. For the transcription reactions, clones pUT17A, pUT27A and pAL3-NcoP3, containing full-length cDNAs of AMV RNA1, RNA2 and RNA3, respectively, were linearized with appropriate restriction enzymes and transcribed with mMESSAGE mMACHINE^®^ T7 kit (Ambion, USA). The quantification of the AMV RNAs was performed with a ND-1000 spectrophotometer (Thermo Scientific, USA) and agarose gel eletrophoresis using an RNA ladder (RiboRuler High Range RNA Ladder 200 to 6000, Thermo Scientific) and several dilutions of the transcribed RNAs.

Before addressing the specific questions of this study, we estimated the minimal amount of AMV transcripts required to initiate an infection in the different hosts by performing serial dilutions of an initial inoculum mixture with a ratio 1:1:1. Henceforth, all ratios of AMV genomic RNA segments are given as RNA1:RNA2:RNA3 (±1 SEMs). For *N. benthamiana* plants we selected a final concentration of total RNAs of 40 ng/μL × 5 μL = 0.2 μg each, whereas for the rest of hosts it was necessary to increment the transcripts concentration five times (200 ng/μL × 5 μL = 1 μg total RNA).

All species were inoculated with the AMV RNAs ratio of 1:1:1 (three plants per ratio) except *N. benthamiana* plants that were also inoculated with ratios: 10:1:1, 1:10:1, 1:1:10, 10:10:1, 10:1:10, and 1:10:10. For each of these experiments, at least three plants were inoculated. All plants were grown in a biosafety level-2 greenhouse at 24/20 °C day/night temperature with 16 h light. After 7 dpi (*N. benthamiana*) or 12 dpi (rest of species), all inoculated plants were analyzed for the abundance of each RNA segment in both total RNA extraction and virus particle purification from inoculated (always the 3^rd^ true leave), 5^th^ and ≥ 8^th^ leaves (Fig. 1A) and from the remaining tissues of the plants (*i.e*, four samples per plant).

### Virus particles purification and total RNA extraction

Leaves (Fig. 1A) or entire plants were homogenized with mortar and pestle in liquid N_2_ to minimize the putative irregular virus distribution in the tissue. Total RNA extraction was performed using 0.1 g of tissue and the Plant RNA Isolation Mini Kit (Agilent, USA) following the manufacturer’s protocol. All samples were diluted to a final concentration of 50 ng of total RNA/µL. Virus particles purification was performed using 0.5 g of the homogenized tissue, following the protocol previously described^57^. The fraction of enriched virus particles was resuspended in 100 μL of PE buffer (10 mM NaH_2_PO_4_, 1 mM EDTA, pH 7.0), that was subsequently subjected to RNA extraction using the Plant RNA Isolation Mini Kit (Agilent, USA). All RNA samples were stored at −80 °C until use.

### Quantification of AMV RNAs by RT-qPCR

The standard curves to quantify the AMV RNA1, RNA2 and RNA3 in the samples by RT-qPCR were prepared using known amounts of DNase-treated transcripts derived from the linearized pUT17A, pUT27A and pAL3-NcoP3 plasmids, respectively. To ensure a correct estimation of the transcripts concentration, all sample were analyzed with a ND-1000 spectrophotometer (Thermo Scientific, USA) and by agarose gel electrophoresis. To construct the standard curve for each RNA, we selected six (RNA2 and RNA3) or seven (RNA1) different viral RNAs concentrations, calculated in terms of molecules/μL (www.endmemo.com/bio/dnacopynum.php), that were generated by 5-fold serial dilutions of a starting solution containing 10^10^ (RNA1) or 2 × 10^9^ (RNA2 and RNA3) molecules of the corresponding viral RNA per μL. All dilutions were made in a solution containing 50 ng/μL of total RNA extracted from healthy *N. benthamiana* plants.

The primers used for amplifying RNA1, RNA2 and RNA3 were designed using PrimerQuest^®^ Design Tool version 2.2.3 (IDT Inc., USA), selecting the parameters GC% = 40–60%, *T*_*m*_ = 57–60 °C, and size = 100–150 bp. The primers used for the RT-qPCR reactions for AMV RNA1, RNA2 and RNA3 are listed in Supplementary Table S1 online. The specificity of each primer set was confirmed with independent reactions using each one of the three RNAs as templates and with an additional reaction in which total RNA extracted from healthy plants was used as template. All these control reactions rendered negative results, except for the appropriate combination of primer and template RNA. To estimate the number of genome equivalents present and their frequencies, all data for the standard curve were first log-transformed to ascertain the range over which the response was linear. The dynamic range was limited to one dilution before the response appeared to saturate. Linear regression of the log-transformed data was then performed, rendering high values for the determination coefficient (*R* ^2^ > 0.98) and of the slope-derived amplification efficiency (90–110%). For those samples that fell within the dynamic range, the estimated linear regression parameters were used to estimate the unknown concentrations in the virus samples.

Duplicated RT-qPCR reactions were carried out in 10 μL reaction volume using the GoTaq^®^ 1-step RT-qPCR system (SYBR^®^ Green) (Promega, USA) and the StepOnePlus Real-Time PCR System (Applied Biosystems, USA). Each reaction contained 50 ng RNA sample, 5 μL of the 2× master mix, 10 μM of both the forward and reverse primer, 0.2 μL of GoScript^TM^ RT Enzyme Mix and 0.155 μL of CXR reference Dye (30 μM). The reactions were incubated at 42 °C for 15 min, followed by 95 °C during 10 min and 40 cycles of 95 °C for 10 s, 62 °C for 34 s and 72 °C for 30 s. After the RT-qPCR reaction, the melting curve stage was determined by incubating 95 °C for 15 s, 60 °C for 1 min and 95 °C for 15 s. The quantification of RNAs 1, 2 and 3 copy number was calculated using the StepOne Software v.2.2.2 (Applied Biosystems, USA).

Supplementary File S2 online contains the absolute quantifications of the three RNA segments for all the experimental samples used in this study.

### Statistical methods

The number of copies of RNA segment *i*, *RNA*_*i*_, on each sample were transformed into relative frequencies, *f*_*i*_, by dividing them by the sum of the values estimated for every RNA segment on the corresponding sample, averaged across the two technical replicates of RT-qPCR: ${f}_{i}=RN{A}_{i}/\sum _{j=1}^{3}\overline{RN{A}_{j}}$. To analyze the effect that different inocula mixtures of the three RNA segments had on the outcome of infection, frequency data shown in Fig. 1B and C were fitted to a multivariate linear model using MANOVA techniques. The model equation fitted reads:

1 $${\overrightarrow{f}}_{ijkl}=\overrightarrow{\phi }+{M}_{i}+P{(M)}_{ij}+S{(M)}_{ik}+(P\times S){(M)}_{ijk}+{\xi }_{ijkl},$$

where ${\overrightarrow{f}}_{ijkl}$ is the vector of frequencies measured for technical replicate *l* ∈ {1,2} of sample *S*_*k*_ (*k* ∈ {inoculated leaf (3^rd^ true leaf), 4^th^ leaf, 5^th^ leaf, and the rest of the plant (stems + apical tissues)}) taken from plant replicate *P*_*j*_ (*j* ∈ {1,2,3}) that was inoculated with a mixture *M*_*i*_ (*i* ∈ {1:1:1, 10:1:1, 1:10:1, 1:1:10, 10:10:1, 10:1:10, 1:10:10}) of RNA segments. Factors *P* and *S*, as well as their interaction, were treated as orthogonal, and nested within factor *M*. ${\xi }_{ijkl}$ measures the experimental error and was assumed to be normally distributed. $\overrightarrow{\phi }$ is the vector of grand mean frequency values and represents a statistical estimate of the *SGF*. Wilk’s Λ distribution was used for the multivariate tests of each factor in the model.

To assess the magnitude of effects we used the ${\eta }_{P}^{2}$ statistic that represents the proportion of total variability attributable to a given factor while controlling for all other factors. The advantage of ${\eta }_{P}^{2}$ respect other measures of effect magnitude is that it allows for comparisons among different experimental designs. Conventionally, values of ${\eta }_{P}^{2}$ < 0.05 are considered as small, 0.05 $\le {\eta }_{P}^{2} <$ 0.15 as medium and ${\eta }_{P}^{2}\ge$ 0.15 as large

To analyze whether *SGF* depends on the host species inoculated with a 1:1:1 mixture, the corresponding frequency data (Fig. 2A and B) were fitted to the following multivariate linear model:

2 $${\overrightarrow{f}}_{ijkl}=\overrightarrow{\phi }+{E}_{i}+P{(E)}_{ij}+S{(E)}_{ik}+(P\times S){(E)}_{ijk}+{\xi }_{ijkl}.$$

In this case, factor *E*_*i*_ represents the plant species (*i* ∈ {*C. annuum*, *C. pepo*, *M. sativa*, *N. benthamiana*, *N. tabacum*} and all other factors are as described for equation (1).

To analyze whether *SGF* is affected by the transgenic expression of viral proteins P1, P2 and P12 in plants inoculated with a 1:1:1 mixture, the estimated segment frequencies (shown in Fig. 4A and C) were fitted to the following multivariate linear model:

3 $${\overrightarrow{f}}_{ijk}=\overrightarrow{\phi }+{E}_{i}+{S}_{ij}+{(E\times S)}_{ij}+{\xi }_{ijk},$$

where *E*_*i*_ now represents the *N. benthamiana* genotype and *i* ∈ {wildtype, P1, P2, P12} and all other factors are as described for equation (2). In this case, only one plant per genotype was assessed.

Next, we considered whether there was frequency-dependent evolution of the ratio of RNA segments infection of plants. In other words, we considered whether the frequency of one segment depends in a positive or negative manner on the abundance of the other two segments. Here, we made use of the classic population genetic approach described by Ayala & Campbell^32^. In short, the ratio of the *j* ^th^ RNA segment to its two counterparts was computed as ${{\rm{\Omega }}}_{j}=RN{A}_{j}/\sum _{k\ne j}RN{A}_{k}$ for both the input mixture and the observed output mixture. In the absence of frequency-dependent selection (FDS), it is expected that the regression of the output ${\mathrm{log}\,{\rm{\Omega }}}_{j}^{o}$ on the input ${\mathrm{log}\,{\rm{\Omega }}}_{j}^{i}$ would be linear with slope one^32^. Significant deviations from the slope one relationship are taken as evidence of positive or negative FDS.

If FDS exists, then it can be evaluated whether (*i*) one or more equilibrium points exist and (*ii*) their stability. If the relationship between ${\mathrm{log}\,{\rm{\Omega }}}_{j}^{o}$ and ${\mathrm{log}\,{\rm{\Omega }}}_{j}^{i}$ is linear, a single equilibrium point exists. If the relationship is not linear, then the number of equilibria equals the number of times the best-fitting function intersects with the diagonal of the ${\mathrm{log}\,{\rm{\Omega }}}_{j}^{o}$ - ${\mathrm{log}\,{\rm{\Omega }}}_{j}^{i}$ phase diagram (*i.e*., the equation of slope one and intercept zero). Equilibria stability can be assessed by evaluating the value of the derivative $d{\mathrm{log}\,{\rm{\Omega }}}_{j}^{o}/d{\mathrm{log}\,{\rm{\Omega }}}_{j}^{i}$ at the corresponding equilibrium point. ${d{\mathrm{log}{\rm{\Omega }}}_{j}^{o}/d{\mathrm{log}{\rm{\Omega }}}_{j}^{i}|}_{eq}$ < 1 corresponds to a stable equilibrium in which the three segments coexist whereas ${d{\mathrm{log}{\rm{\Omega }}}_{j}^{o}/d{\mathrm{log}{\rm{\Omega }}}_{j}^{i}|}_{eq}$> 1 corresponds to the case of a non-stable one in which the abundances of the three segments may experience changes due to very small perturbations.

In all cases, segment frequency data obtained from total RNA extractions and from virus preparations were analyzed separately. MANOVA and other statistical analyses were done using IBM SPSS version 23 (Armonk, NY, USA).

## Electronic supplementary material

Table S1

Supplementary information
