# Supplementary material for: Within-host Evolution of Segments Ratio for the Tripartite Genome of Alfalfa Mosaic Virus
Source: Sci Rep. 2017 Jul 10;7:5004. doi: 10.1038/s41598-017-05335-8 (PMC5504059; doi:10.1038/s41598-017-05335-8)
Supplement: Supplementary file 1 — Table S1 [file 41598_2017_5335_MOESM1_ESM.pdf]

Within-host Evolution of Segments Ratio for the Tripartite Genome of *Alfalfa Mosaic Virus*

Beilei Wu, Mark P. Zwart, Jesús A. Sánchez-Navarro & Santiago F. Elena

Supplementary Table S1. Primers used to amplify each RNA segment.

RNA	Primers name and sense	Range	Sequence (5'→3')
RNA1	2796sAMVRNA1 forward	1300-1319	CGGTGGTGTGACTCTGATTT
	2797AsAMVRNA1 reverse	1391-1412	CTCCCTTGTCTAGGAGGGATAA
RNA2	2798sAMVRNA2 forward	664-685	GAGGAACTTGGACCTCTGAATG
	2799AsAMVRNA2 reverse	727-748	TGTCGAGTGGCTTAGCATTATC
RNA3	2800sAMVRNA3 forward	606-625	CGTCCATCACTCGGCTATTT
	2801AsAMVRNA3 reverse	685-705	TCGGTGTCAACATCCACTAAC
